# Reelin promotes the adhesion and drug resistance of multiple myeloma cells via integrin β1 signaling and STAT3

**DOI:** 10.18632/oncotarget.7151

**Published:** 2016-02-03

**Authors:** Liang Lin, Fan Yan, Dandan Zhao, Meng Lv, Xiaodong Liang, Hui Dai, Xiaodan Qin, Yan Zhang, Jie Hao, Xiuyuan Sun, Yanhui Yin, Xiaojun Huang, Jun Zhang, Jin Lu, Qing Ge

**Affiliations:** ^1^ Key Laboratory of Medical Immunology, Ministry of Health, Department of Immunology, School of Basic Medical Sciences, Peking University Health Science Center, Beijing 100191, China; ^2^ Peking University Institute of Hematology, People's Hospital, Beijing 100044, China; ^3^ Department of Immunology, Tianjin Medical University Cancer Institute and Hospital, Tianjin 300060, China; ^4^ Jining No.1 People's Hospital, Jining, Shandong 272011, China; ^5^ Hangzhou Cancer Hospital, Hang Zhou 310002, China

**Keywords:** multiple myeloma, reelin, adhesion, integrin, STAT3

## Abstract

Reelin is an extracellular matrix (ECM) protein that is essential for neuron migration and positioning. The expression of reelin in multiple myeloma (MM) cells and its association with cell adhesion and survival were investigated. Overexpression, siRNA knockdown, and the addition of recombinant protein of reelin were used to examine the function of reelin in MM cells. Clinically, high expression of reelin was negatively associated with progression-free survival and overall survival. Functionally, reelin promoted the adhesion of MM cells to fibronectin via activation of α5β1 integrin. The resulting phosphorylation of Focal Adhesion Kinase (FAK) led to the activation of Src/Syk/STAT3 and Akt, crucial signaling molecules involved in enhancing cell adhesion and protecting cells from drug-induced cell apoptosis. These findings indicate reelin's important role in the activation of integrin-β1 and STAT3/Akt pathways in multiple myeloma and highlight the therapeutic potential of targeting reelin/integrin/FAK axis.

## INTRODUCTION

The strong and complex interactions between multiple myeloma (MM) cells and the microenvironment of the bone marrow (BM) lead to tumor cell survival, proliferation, invasion, and the development of acquired drug resistance [1-2]. For example, the activation of integrin β1 promotes MM cell adhesion to BM extracellular matrix (ECM) proteins, including laminin, microfibrillar collagen type VI, and fibronectin (FN). Such adhesion could induce upregulation of antiapoptotic bcl-2 family members and/or overexpression of *multidrug resistant gene 1*, thereby causing malignant cells to become unresponsive to anticancer drugs, a phenomenon called “cell adhesion-mediated drug resistance” (CAM-DR) [3-13]. Thus, the modulation of MM cell adhesion via blocking of integrin β1 resulted in beneficial therapeutic effects when combined with chemotherapy [6, 14-15].

The ECM protein reelin has been found in the brain [16-20] and several types of peripheral tissues and cells [21-23]. Certain types of tumor also up-regulate reelin expression, including high Gleason score prostate cancer, esophageal carcinoma, and retinoblastoma [24-27]. Extensive studies have shown that reelin plays an essential role in regulating the correct migration and positioning of cortical neurons and spines [16-20]. However, how reelin affects the adhesion, migration, and survival of tumor cells remains unclear. To understand the contribution of reelin to cancer pathology, we investigated its expression and function in MM cells.

## RESULTS

### High RELN expression negatively correlates with progression-free survival and overall survival in MM patients

**Figure 1:**
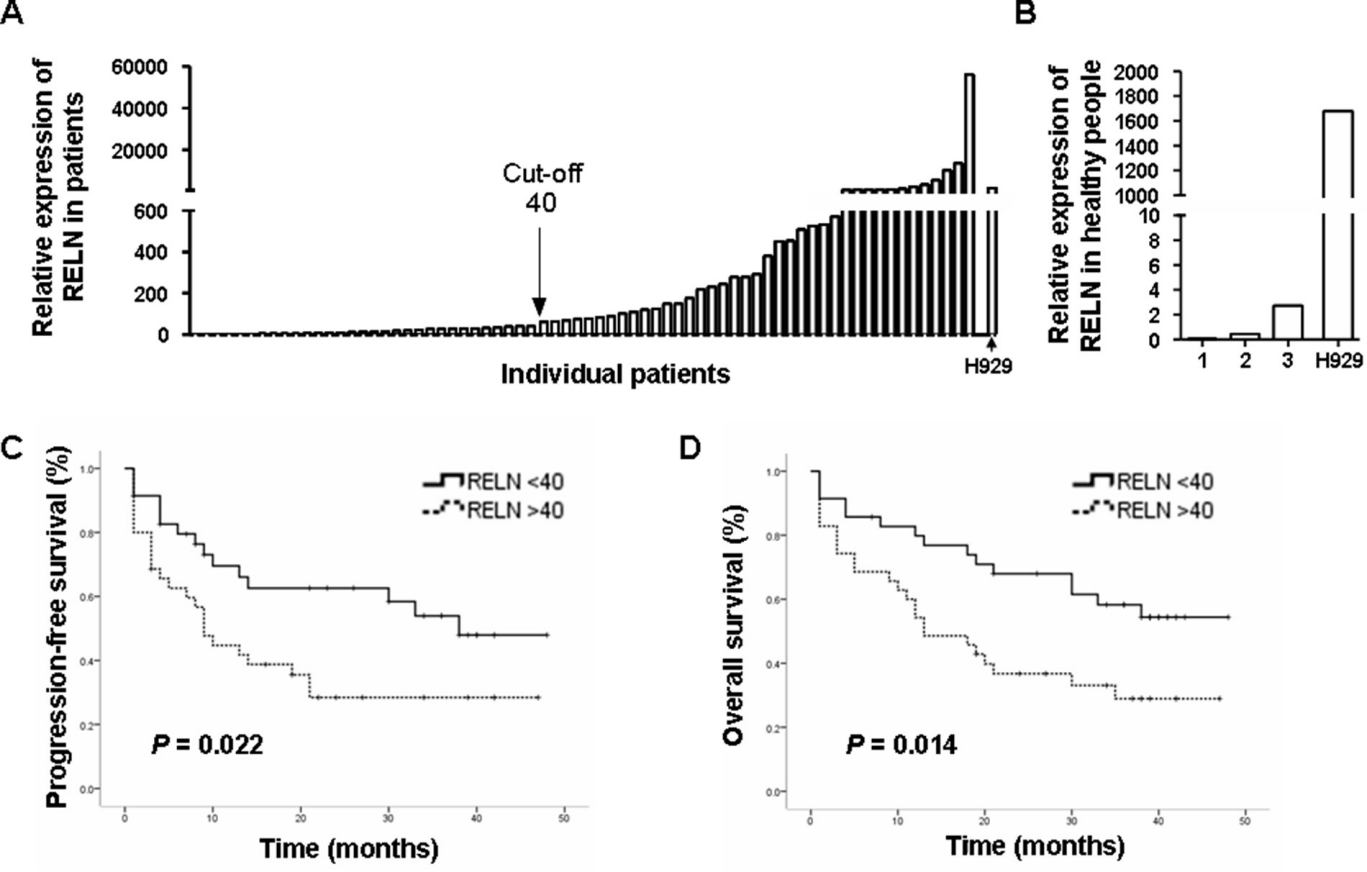
*RELN* expression is negatively associated with PFS and OS in MM patients **A.** The relative *RELN* expression among MM patients. CD138^+^ MM cells were purified from bone marrow by flow cytometry. Total RNAs were then extracted and real time PCR was performed to measure the level of RELN transcription. The quantification was based on ΔΔCT calculations and was normalized to GAPDH as a housekeeping gene control. *RELN* expression in one of the MM cell lines, H929, was used as an internal control. **B.** The relative RELN expression of CD138^+^ bone marrow cells among three healthy donors. **C.** Association of *RELN* expression with progression-free survival (PFS). **D.** Association of *RELN* expression with overall survival (OS).

To determine the expression of *RELN* in multiple myeloma, CD138^+^ cells from the BM aspirates of 3 healthy donors and 70 newly diagnosed or relapsed MM patients were purified and subjected to RNA extraction and quantitative RT-PCR (Figure 1A and Supplemental Figure 1A). *RELN* expression in one of the MM cell lines, H929, was used as an internal control and GAPDH was used as a housekeeping gene control. The CD138^+^ cells from healthy donors exhibited very low level of *RELN* expression (Figure 1B). In patients, various amounts of *RELN* was found in CD138^+^ myeloma cells and a hierarchical cluster analysis with Ward's method was used to analyze the relative expression fold of *RELN* (compared with the GAPDH control). An arbitrary cut-off value was then set at 40-relative expression fold to separate low from high *RELN* expression. The group with low *RELN* expression had better progression-free survival (PFS) and overall survival (OS) than that with high *RELN* expression (Figure 1C-1D). The Median PFS for low and high RELN expression groups were 30 months (95% confidence interval (CI): 23.7, 37.3) and 19 months (95% CI: 12.3, 25.0), respectively (*P* = 0.022). The OS for low and high *RELN* groups were 34 months (95% CI: 27.6, 39.6) and 21 months (95% CI: 15.3, 27.6), respectively (*P* = 0.014). In addition, high *RELN* expression was associated with higher numbers of tumor cells in the bone marrow (42.0% ± 24.9% for high *RELN* and 28.5% ± 22.8% for low *RELN* expressions, *P* = 0.029). No significant association was found between *RELN* expression and extramedullary disease (EMD), with 11% EMD in the low *RELN* group and 23% in the high *RELN* group, *P* = 0.205. These results suggest that reelin may facilitate MM progression in the BM.

### Reelin promotes MM cell adhesion to ECM

**Figure 2:**
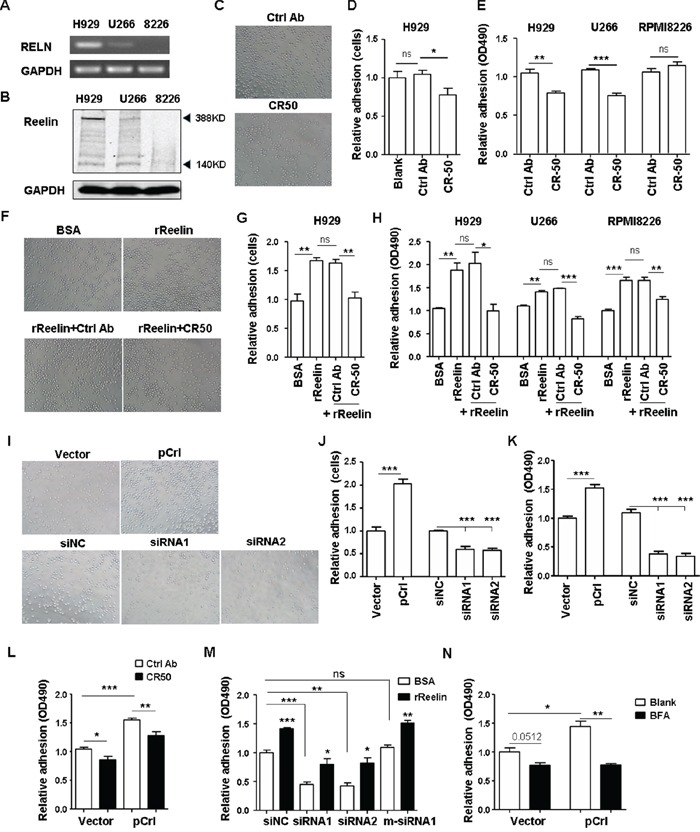
Reelin promotes the adhesion of HMCLs to FN The expressions of reelin mRNA **(A.)** and protein **(B.)** in H929, U266, and RPMI8226 cells were assessed by RT-PCR (A) and western blotting (B). **C-E.** Reelin blockage decreases HMCL adhesion to FN. H929, U266, and RPMI8226 cells were treated with reelin blocking antibody CR-50 before seeded into FN-coated plates. The attached cells were analyzed by light microscopy (C), by calculating the average cell numbers per well (D) or by colorimetric cell adhesion assay (E). The values obtained from CR-50 treated samples were calculated relative to the samples untreated (Blank) or treated with isotype control (Ctrl) antibody and were shown as relative adhesion (cells) in (D) or relative adhesion (OD490) in (E). **F-H.** Pre-incubation of recombinant reelin (rReelin) alone increases whereas rReelin with blocking antibody CR-50 decreases the adhesion of HMCLs to FN. The attached cells analyzed by light microscopy were shown in (F), calculated by the average cell numbers per well were shown in (G) or by colorimetric cell adhesion assay in (H). The values obtained from FN-coated wells were calculated relative to the wells coated with BSA and were shown as relative adhesion (cells) in (G) or relative adhesion (OD490) in (H). **I-K.** Overexpression or knockdown of reelin changes the adhesion of HMCLs toward FN. H929 cells were transfected with pCrl or reelin-specific siRNA. Cell adhesion to FN-coated plates was then analyzed by light microscopy (I), by average cell numbers per well (J), and by colorimetric analysis (K). **L-M.** The promotion of H929 cell adhesion to FN is reelin specific. H929 cells transfected with pCrl or pcDNA3 were cultured in FN-coated plates with CR-50 or isotype control (L). H929 cells transfected with RELN-specific siRNAs or m-siRNA1 were cultured in FN-coated plates in the presence or absence of recombinant reelin (M). Cell adhesion was analyzed by colorimetric cell adhesion assay. **N.** Reelin secretion is required for the promotion of MM cell adhesion. The pCrl-transfected cells were treated with BFA for 4 hours before being added to FN-coated wells for adhesion assay. The results are representative of three independent experiments. Error bars indicate the standard deviation. *p<0.05, **p<0.01, ***p<0.005.

To examine the role of reelin in MM pathology, three human myeloma cell lines (HMCLs) were used: H929, RPMI8226, and U266. Among these cell lines, H929 displayed the highest, whereas RPMI8226 displayed the lowest, levels of reelin mRNA and protein (Figure 2A-2B, Supplemental Figure 1B). As shown in Figure 2B, two reelin immunoreactive bands (full length isoform of 388 KDa and a cleaved fragment of 140 KDa [28]) were revealed with the 388 KDa as the major form of reelin protein in HMCL lysates.

As reelin plays an important role in regulating the positioning of neurons, we first investigated whether suppressing intrinsic reelin activity by the addition of a function-blocking anti-reelin antibody (CR-50 [29]) could alter MM cell adhesion to FN. Both adherent cell counting and colorimetric analysis were used to measure cell adhesion. As shown in Figure 2C-2E, CR-50 suppressed the cell adhesion in reelin^hi/int^ H929 and U266 cells but not in reelin^lo^ RPMI8226 cells (the control antibody did not show suppression). To examine whether the adhesion of reelin^lo^ RPMI8226 cells could be improved by reelin, the cells were pre-incubated with recombinant reelin (rReelin) in the presence or absence of CR-50 for an hour. The cells were then thoroughly washed and were tested for their adhesion in FN-coated plates. Reelin^int/hi^ U266 and H929 cells were also included in the experiments. Significantly enhanced cell adhesion was found in all three rReelin-treated HMCLs and was abolished in CR-50-treated ones (Figure 2F-2H). These indicate that reelin can promote MM adhesion to FN.

The involvement of reelin in MM adhesion was further examined by RELN overexpression using the pCrl plasmid (Supplemental Figure 1C-1F) and by knockdown of intrinsic expression using reelin-specific siRNAs (Supplemental Figure 1G-1I). In pCrl-transfected HMCLs, a significant increase in adhesion to FN was observed (Figure 2I-2L for H929 cells and Supplemental Figure 1J for U266 cells). The addition of CR-50 suppressed the adhesion (Figure 2L). In siRNA-transfected H929 cells, however, a significant reduction of adhesion was found and the addition of recombinant reelin protein partially alleviated the inhibition of adhesion (Figure 2I-2K, 2M). When mutations around the siRNA cleavage site were introduced into reelin-specific siRNA (m-siRNA1, Supplemental Figure 1I), the knockdown of reelin expression and the reduction of cell adhesion to FN were both abolished (Figure 2M).

In addition, the adhesion-promoting effect of reelin was completely nullified when Brefelin A was used to block protein transport from the endoplasmic reticulum to Golgi complex (Figure 2N), suggesting that cell-FN adhesion promoted by reelin requires reelin to be released to the extracellular space. We further examined whether the adhesion enhanced by reelin is FN-specific and found that MM cell adhesion to poly-L-lysine (PLL)-coated wells was not increased by the addition of rReelin (Supplemental Figure 1K). No direct adhesion of HMCLs to rReelin was observed, even though reelin itself is an ECM protein (Supplemental Figure 1L). Taken together, these results indicate that reelin expressed and secreted by MM cells specifically promotes MM cell adhesion to FN, probably via the alteration of FN receptors expressed on the cell surface.

### Reelin promotes the drug-resistance of MM cells

**Figure 3:**
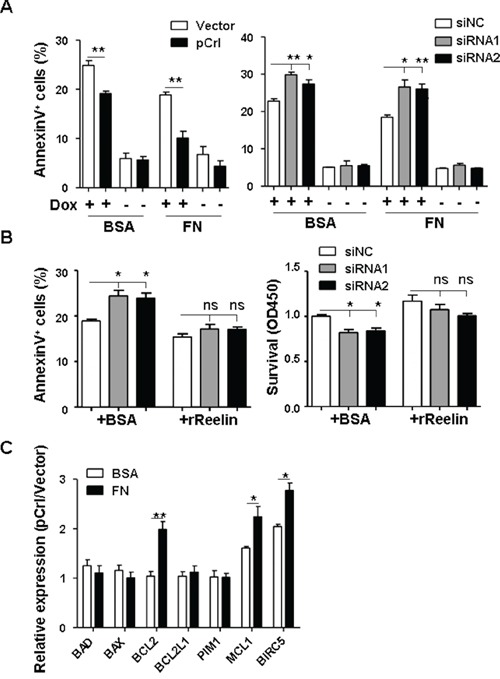
Reelin protects H929 cells from Doxorubicin induced apoptosis **A-B.** H929 cells were transfected with pCrl or reelin-specific siRNAs for 40 hours. The cells were then incubated with or without Dox (2μM) in 5% BSA- or 40 μg/ml of FN-coated plates (A). Alternatively, recombinant reelin or BSA control were added together with Dox into the culture of H929 cells transfected with reelin-specific siRNAs (B). Cell apoptosis was analyzed 24 hours later by annexin V staining and CCK8 method. **C.** Reelin induces the upregulation of anti-apoptotic genes in Dox-treated H929 cells. H929 cells were transfected with pCrl or control vector for 40 hours. The cells were then transferred into 5% BSA- or FN-coated plates in triplicate and cultured in the presence of Dox. Twenty-four hours later, the cells were harvested for quantitative RT-PCR analysis of anti-apoptotic and pro-apoptotic gene expression. ΔΔCt of each sample was calculated and the results were shown as the ratio of 2^ΔΔCt(pCrl)^ to 2^ΔΔCt(vector)^. Data are representative of at least three independent experiments.

The reelin's promotion of MM cell adhesion to FN and the association of high *RELN* expression with poor prognosis led us to examine whether reelin could enhance CAM-DR. H929 cells with altered reelin expression were treated with Doxorubicin (Dox) in the presence and absence of FN. As shown in Figure 3A and Supplemental 2A-2B, the overexpression of reelin significantly protected H929 from Dox-induced cell apoptosis (IC_50_ for reelin overexpression and control samples were 1.6 and 1.1 μM, respectively, Supplemental Figure 2B). The presence of FN further enhanced the protective effect of reelin (IC_50_ for reelin overexpression and control were 4.1 and 3.4 μM, respectively, Supplemental Figure 2B). In contrast, suppressing the intrinsic expression of reelin using specific siRNAs induced more MM cell apoptosis upon Dox treatment, whereas the addition of recombinant reelin alleviated the siRNA's inhibitory effect on Dox-treated MM cells (Figure 3A-3B, Supplemental Figure 2A). Similarly, reelin overexpression protected HMCLs from cisplatin (DDP, Supplemental Figure 2C)- and imatinib mesylate (IM, Supplemental Figure 2D)-induced apoptosis in FN-coated plates. IC_50_ for reelin overexpression and control samples were 40 and 50 μM for DDP, 34 and 40 μM for IM, respectively. In the absence of these agents, reelin overexpression or silencing did not significantly affect H929 cell survival (Figure 3A). These results suggest that reelin promotes MM cell survival.

To examine whether reelin promotes the survival of Dox-treated MM cells via alteration of the balance between pro- and anti-apoptotic gene expressions, *RELN*-overexpressing H929 cells were treated with Dox in the presence and absence of FN for 24 hours before being harvested for quantitative RT-PCR analysis. Compared with the vector control, pCrl-transfected cells had significantly higher expression levels of anti-apoptotic genes such as *BCL2*, *MCL1*, and *BIRC5* (survivin) in the presence or even in the absence of FN, thus tilting the balance of reelin^hi^ MM cells toward cell survival (Figure 3C).

### Reelin promotes the activation of integrin β1

**Figure 4:**
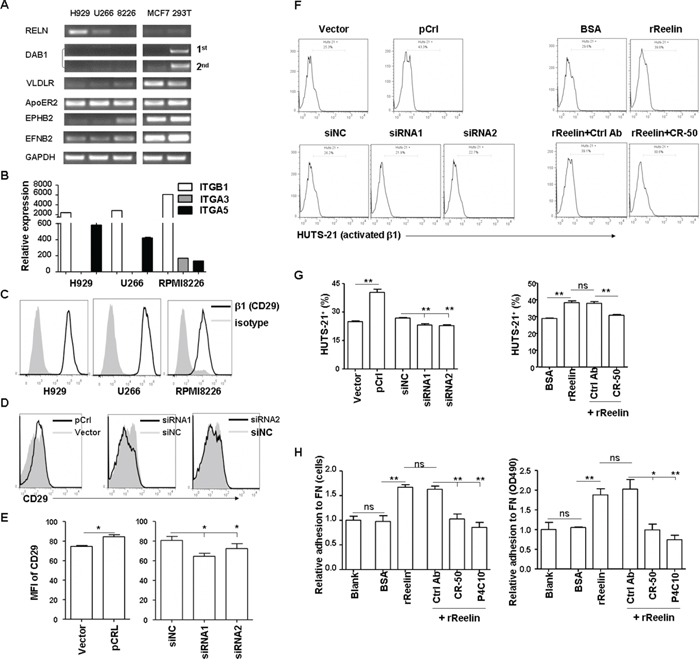
Reelin promotes integrin β1 activation and MM cell adhesion **A.** The expression of reelin binding proteins and adaptor proteins (ApoER2, VLDLR, DAB1, EPHB2, and EFNB2) in HMCLs was analyzed by quantitative RT-PCR. MCF7 and 293T cells were used as controls. **B.** Comparison of integrin α3, α5, and β1 mRNA expressions in HMCLs by quantitative RT-PCR. **C.** Comparison of the surface expression of integrin β1 (CD29) in HMCLs by flow cytometry. **D-E.** Altered reelin levels slightly change the surface expression of CD29 in H929 cells. H929 cells were transfected with pCrl or siRNAs and the expression of CD29 was analyzed by flow cytometry 40 hours later. The histogram obtained from pCrl-transfected cells was overlaid with that from control vector-transfected cells and the histogram obtained from reelin-specific siRNA-transfected cells was overlaid with that from control siRNA-transfected cells (D). Mean fluorescence intensities (MFI) of CD29 from three independent experiments were shown in (E). **F-G.** Reelin induces the activation of integrin β1. pCrl- or siRNA-transfected H929 cells, or plain H929 cells pre-incubated with the rReelin, BSA, rReelin with CR-50, or rReelin with control antibody were cultured in FN-coated plates for 1 hour and were analyzed for the activation of integrin β1 (HUTS-21) by flow cytometry. The percentages of HUTS-21^+^ cells with different treatments were compared (F). Three independent experiments were performed and the mean values and standard deviation are shown (G). **H.** The reelin-induced H929 adhesion to FN is blocked by CR-50 and P4C10. H929 cells were cultured in FN-coated plate in the presence of rReelin, BSA, rReelin with CR-50, rReelin with P4C10, or rReelin with control antibody for 1 hour. The adhesion of these cells to FN was then analyzed by calculating the average cell numbers (left panel) or by colorimetric cell adhesion assay (right panel). Data are representative of three independent experiments.

We next investigated the cell surface molecules that might be involved in reelin-promoted MM cell adhesion. The very low density lipoprotein receptor (VLDLR) and apolipoprotein E receptor 2 (ApoER2) are well-studied high affinity receptors for reelin [30-31]. The binding of these two receptors with reelin results in their interaction with the adaptor protein, Disabled 1 (Dab1), and subsequent phosphorylation of Dab1 by Src family tyrosine kinases (SFKs) [32]. The transmembrane proteins Ephrin B and EphB were reported recently to bind to reelin and subsequently activate the EphB forward signaling [33] or promote the phosphorylation of Dab1 [34]. Thus, the mRNA expressions of these molecules were examined in all three HMCLs. The breast cancer cell line MCF7 and human embryonic kidney 293T cells were used as controls. As shown in Figure 4A, all the HMCLs expressed ApoER2, but very little VLDLR. Ephrin B2 was expressed by all three HMCLs while EphB2 was only expressed in RPMI8226 cells. However, the expression of Dab1 was not detected, even using nested PCR (Figure 4A), suggesting that the activation of Dab1 and its downstream signaling pathway may not be involved in MM cell adhesion promoted by reelin.

We then examined whether the expression and activation of integrin was altered following *RELN* changes. Integrin α3β1 binds directly with reelin, although its effect on neuronal migration or neuron-ECM adhesion remains controversial [18, 35-37]. Reelin can also bind to integrin α5β1, albeit with lower affinity when compared with ApoER2 [38]. As shown in Figure 4B-4C, all three HMCLs expressed α5β1, whereas only RPMI8226 expressed both α3β1 and α5β1. Slightly increased expressions of α5 and β1 mRNAs (Supplemental Figure 3A-3B) and proteins (CD29, Figure 4D-4E) were found in H929 cells after their adhesion to FN or transfection with reelin-expressing plasmid (pCrl). In contrast, reduced mRNA levels of α5 and β1, as well as the protein level of β1 (Figure 4D-4E, Supplemental Figure 3C) were observed in H929 cells transfected with *RELN*-specific siRNAs. Reelin not only increases integrin β1 expression, but also activates it. A reduction in the activated form of β1 (HUTS-21) was observed in H929, U266, and RPMI8226 cells receiving *RELN*-specific siRNAs, whereas an increase in activated β1 was shown in cells receiving pCrl plasmid or rReelin protein (Figure 4F-4G and Supplemental Figure 3D-3F). The addition of CR-50 blocked β1 activation by rReelin (Figure 4F-4G). In addition, Brefeldin A, which blocks the secretion of reelin, also diminished the activation of integrin β1 (HUTS-21) induced by *RELN* overexpression, suggesting that reelin is required to be released from the cells to promote integrin β1 activation (Supplemental Figure 3G).

To test directly whether reelin-induced MM cell adhesion to FN was mediated by β1 integrin, MM cells were treated with the β1 inhibitory antibody P4C10 and rReelin for 1 hour. Similar to the effect of reelin-blocking antibody CR-50, P4C10 also abolished reelin's capability of promoting MM adhesion to FN (Figure 4H). These data suggest that reelin likely enhances the binding capacity of the extracellular domain of integrin α5β1 to their ligands in ECM (integrin activation), thereby promoting MM cell adhesion.

### Phosphorylation of FAK, Src, and Akt contributes to reelin/β1-induced cell adhesion

**Figure 5:**
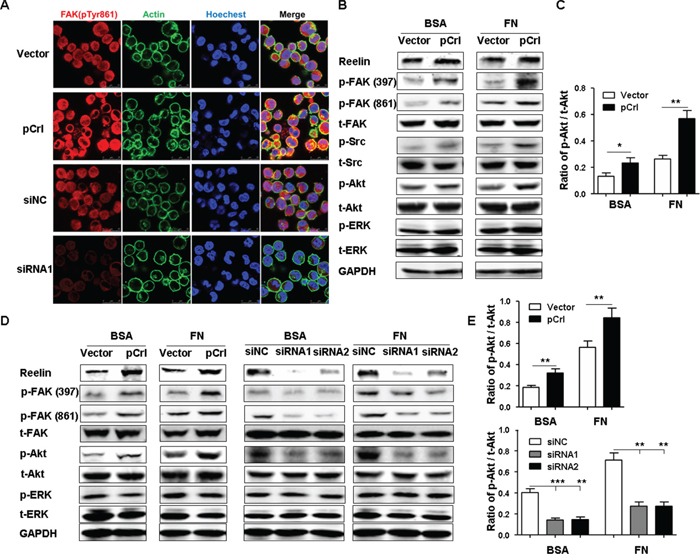
Reelin promotes integrin β1 signaling via activation of FAK, Src, and Akt **A.** Alteration of reelin expression in H929 cells changes the phosphorylation of FAK at Tyr 861. H929 cells were transfected with reelin expressing plasmid pCrl or control plasmid, or reelin-specific or control siRNA for 40 hours. The cells were cultured in FN-coated plates overnight, and stained with antibodies against phospho FAK Tyr 861 (red), actin (green). Nuclei were stained with Hoechest 33342. The cells were analyzed by laser-scanning confocal microscopy (160x). **B.** The activation of FAK, Src, and Akt by reelin. H929 cells transfected with pCrl or siRNAs were cultured in the presence or absence of FN. One hour later, the cells were harvested and cell lysates were subjected to western blotting with FAK, Src, Akt, ERK1/2 antibodies and FAK Tyr397 (Y397), FAK Tyr861, Src Tyr416, Akt Ser473, ERK1/2 Tyr202/Tyr204 phospho-specific antibodies. An antibody specific for GAPDH was used as loading control. **C.** Densitometric quantification of the expression ratio of phospho-Akt (Ser473) over total Akt from the blot shown in (B). The mean ± SD of three experiments was shown. **D.** Reelin promotes FAK and Akt activation in H929 cells upon Doxorubicin treatment. H929 cells were transfected with pCrl or reelin-specific siRNAs for 40 hours and then treated with Dox in the presence of 5% BSA or FN. Twenty-four hours later, the cells were harvested and cell lysates were subjected to western blotting for the activation of FAK, Akt, and ERK1/2. **E.** Densitometric quantification of phospho-Akt over total Akt from the blot shown in (D). Data are representative of at least three independent experiments.

To examine if reelin activates the integrin β1 signaling pathway, the phosphorylation of focal adhesion kinase (FAK) and Src, members of integrin complexes and key mediators of signaling downstream of integrins [39], were measured. H929 transfected with pCrl or specific siRNAs were cultured in FN-coated slides overnight and were examined for the phosphorylation of tyrosine-861 (Tyr861) of FAK by confocal microscopy. As shown in Figure 5A, the activation of FAK was enhanced in the cells overexpressing reelin and reduced in the cells with reelin knock-down (also shown in Supplemental Figure 4A). Western blotting also showed that pCrl-transfected MM cells had significantly more phosphorylation of FAK at both Tyr397 and Tyr861 than control vector-transfected ones in the presence of FN (Figure 5B). A slightly more FAK phosphorylation at Tyr861 was also found in H929 cells transfected with pCrl in the absence of FN (BSA group) (Figure 5B). The total amount of FAK did not appear to change. A comparison of Src phosphorylation between pCrl- and control vector-transfected H929 cells also revealed that the phosphorylation of Src was slightly enhanced in reelin-overexpressing cells in the absence of FN and was further enhanced in the presence of FN. As FAK/Src has been shown to activate phosphoinositide 3-kinase (PI3K) and Akt to promote cell adhesion and survival [40-41], the phosphorylation of Akt at Ser-473 was examined. H929 cells transfected with pCrl in FN-coated wells showed a slight but significant increase in Akt phosphorylation when compared to controls (Figure 5B-5C). The second pathway activated by FAK/Src, the ERK pathway, was also examined. No differences of ERK1/2 phosphorylation were found between pCrl- and control vector-transfected cells (Figure 5B). Similar results were also found in U266 cells (Supplemental Figure 4B). These data indicate that the activation of integrin β1 by reelin results in the phosphorylation of the FAK/Src/Akt pathway.

To determine if reelin-induced refractoriness of MM cells to Dox is associated with reelin's activation of FAK/Src/Akt pathway, H929 cells transfected with pCrl or reelin-specific siRNAs were treated with 2 μmol/L Dox in the presence or absence of FN. Twenty-four hours later, the cells were harvested and subjected to western blotting. Compared with their respective controls, more phosphorylation of FAK and Akt was found in pCrl-transfected cells, whereas less activation of these signaling molecules was found in reelin-specific siRNA-transfected ones (Figure 5D-5E). Again, the phosphorylation of ERK was not affected by altering reelin expression levels in H929 cells. Notably, the differences in FAK and Akt activation were also seen in H929 cells in the absence of FN, implying that the activation of FAK/Src/Akt pathway by reelin may not completely require activated integrin β1 to bind with FN.

### Phosphorylation of Syk and STAT3 contributes to reelin/β1-induced cell adhesion and CAM-DR

**Figure 6:**
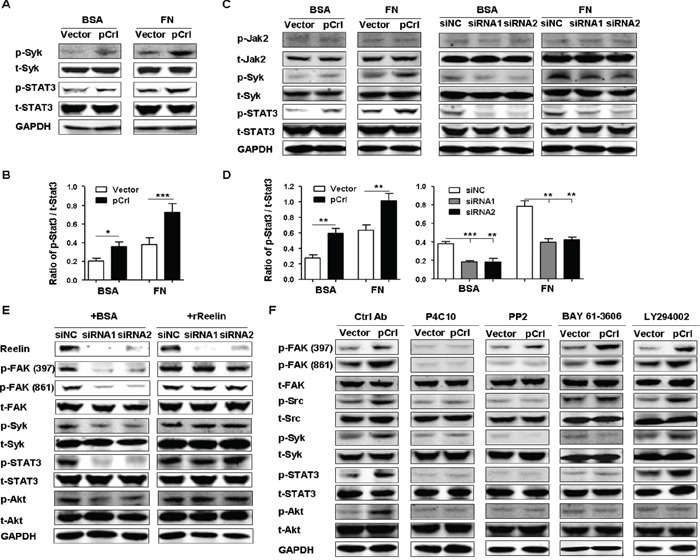
Reelin promotes the activation of Syk and STAT3 via integrin β1 **A.** Reelin induces the activation of Syk and STAT3. H929 cells transfected with pCrl or control plasmid were cultured in 5% BSA- or FN-coated plates for 1 hour. The cells were then subjected to western blotting with phospho-STAT3 (Tyr705), total STAT3, phospho-Syk (Tyr525/526), and total Syk-specific antibodies. **B.** Densitometric quantification of phospho-STAT3 over total STAT3 from the blot shown in (A). **C.** Reelin promotes activation of Syk and STAT3 in H929 cells treated with Doxorubicin. H929 cells were transfected with pCrl or reelin-specific siRNAs for 40 hours and were then treated with Dox (2 μM) in the presence or absence of FN. Twenty-four hours later, the cells were subjected to western blotting for the activation of Jak2 (phospho-Jak2, Tyr1007/1008), Syk, and STAT3. **D.** Densitometric quantification of phospho-STAT3 over total STAT3 from the blot shown in (C). **E.** The reduced activation of FAK, Src, Syk, and STAT3 caused by reelin-specific siRNAs is ameliorated by the addition of recombinant reelin. Reelin-specific siRNA-transfected H929 cells were treated with Dox in FN-coated wells in the presence of BSA or recombinant reelin. Cell harvesting and western blotting was performed 24 hours later. **F.** The effects of integrin β1 inhibitory antibody, Src inhibitor, Syk inhibitor and PI3K inhibitor on the activation of β1 signaling pathway. H929 cells were transfected with pCrl or control plasmid. Forty hours later, the cells were cultured in FN-coated plates and were treated with integrin β1 inhibitory antibody P4C10, Src inhibitor PP2, Syk inhibitor BAY 61-3606, or PI3K inhibitor LY 294002 for 24 hours. The cells were then lysed and subjected to western blotting. Data are representative of three independent experiments.

The anti-apoptotic molecules Bcl-2, Mcl-1, and survivin that are upregulated by reelin overexpression are targets of signal transducer and activator of transcription (STAT)3. Therefore, we examined whether reelin/integrin β1 could activate this oncogenic transcription factor. Compared with the vector control, a slight increase in STAT3 phosphorylation at Tyr-705 was already seen in pCrl-transfected H929 cells without FN (Figure 6A-6B). The same cells further enhanced STAT3 activation when FN was present (Figure 6A-6B). The total amount of STAT3, however, did not change.

The phosphorylation of STAT3 by integrin β2 was reported recently in acute myeloid leukemia [42] and cutaneous T-lymphoma [43]. The integrin-activated spleen tyrosine kinase (Syk) was shown to promote STAT3 phosphorylation [42, 44]. To determine whether Syk activation is also associated with reelin-induced β1 activation and STAT3 phosphorylation, the phosphorylation of Syk at Tyr525/526 was examined in reelin-overexpressing H929 cells. Similar to the results of STAT3, more Syk phosphorylation was seen in pCrl-transfected cells than in control cells in the presence of FN (Figure 6A). Furthermore, upon Dox treatment, H929 cells with pCrl-transfection showed enhanced activation of Syk and STAT3, whereas the cells with reelin-specific siRNA transfection exhibited decreased phosphorylation of these two molecules (Figure 6C-6D). Similar results were obtained in pCrl-transfected U266 cells (Supplemental Figure 4C). In addition, enhanced FAK, Src, and STAT3 activation could be also observed in U266 and RPMI8226 cells when treated with rReelin (Supplemental Figure 4D-4E).

A previous report indicated that the concomitant exposure of MM cells to IL-6 and FN adhesion increased STAT3 phosphorylation [45]. We thus determined if reelin activates STAT3 via IL-6 induction. However, neither IL-6 production nor JAK2 phosphorylation were altered following reelin-expressing plasmid or siRNA transfection (Figure 6C and Supplemental Figure 4F-4G). In addition, the decrease in FAK, Syk, STAT3, and Akt activation in cells transfected with reelin-specific siRNA was abolished when these cells were replenished with recombinant reelin protein (Figure 6E), confirming a role of STAT3 activation in reelin/integrin pathway. We further examined if reelin-induced CAM-DR of MM cells is mediated by STAT3. As shown in Supplemental Figure 4H-4J, STAT3-specific siRNAs abolished reelin's effect on promoting the survival of Dox-treated H929 cells. But FN adhesion of H929 cells transfected with pCrl was not decreased by STAT3 siRNAs.

We next used inhibitory antibodies or chemicals against these signaling molecules to further examine the signaling pathways activated by reelin. Upon treatment of H929 cells with β1 inhibitory antibody P4C10, the enhanced phosphorylation of FAK and the subsequent activation of Src, Syk, STAT3, and Akt disappeared in reelin-overexpressing cells, confirming the activation of integrin β1 signaling by reelin (Figure 6F). The addition of Src kinase inhibitor PP2 to the pCrl-transfected cells resulted in a strong decreases in Src, Syk, STAT3 and Akt activation (Figure 6F). As the maximal activation of FAK, including the phosphorylation of Tyr861, depends on the recruitment and activation of Src, the phosphorylation of FAK Tyr861, but not Tyr397, was also suppressed by PP2 (Figure 6F). The Syk inhibitor, BAY61-3606, suppressed the activation of Syk, STAT3 and Akt, whereas the phosphorylation of the upstream molecules FAK and Src were not inhibited (Figure 6F), suggesting that reelin-induced Syk phosphorylation is a result of FAK/Src activation and the subsequent activation of STAT3 and Akt requires Syk phosphorylation. The treatment of H929 cells with LY294002, an inhibitor of phosphoinositide 3-kinase (PI3K), decreased the activation of Akt, but not that of FAK, Src, Syk, and STAT3 (Figure 6F). Taken together, these results indicate that reelin promotes the adhesion and CAM-DR of MM cells by activating integrin β1/FAK and its downstream Src-Syk-STAT3/Akt pathway.

## DISCUSSION

Altered reelin expression has been found in many types of tumors [24-26, 46-50]. Except for a pancreatic cancer cell line that showed the involvement of the well-studied reelin receptors ApoER2/VLDLR and their key adaptor protein, Dab1, in the suppression of cell migration by reelin [46], none of the studies have investigated reelin's functions and signaling pathways in tumorigenesis. In this study, we show that reelin produced by MM cells promotes the adhesion of tumor cells to fibronectin via activation of integrin β1-FAK pathway and the subsequent activation of Akt and STAT3 signaling molecules, eventually facilitating the survival and drug resistance of myeloma cells.

The activation of integrin β1 signaling pathways regulates a variety of tumor cell functions [51-52]. Specifically, the engagement and clustering of integrins by reelin allows FAK Tyr397 autophosphorylation. The maximal activation of FAK, including the phosphorylation of Tyr861, depends on the recruitment and activation of Src. Thus, a Src inhibitor could suppress the phosphorylation of FAK Tyr861 induced by reelin (Figure 6D). Phosphorylated FAK could also activate ERK1/2 via FAK/Ras GTPase/Raf1/MEK1/ERK1/2 pathway [39]. However, we did not observe an increase in ERK1/2 phosphorylation upon reelin-induced integrin activation, suggesting that this pathway may not play an essential role in reelin/integrin β1 signaling.

**Figure 7:**
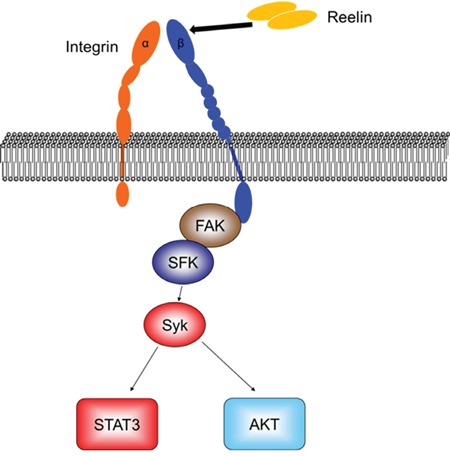
Diagram of reelin-induced signaling pathways in myeloma cells

The activation of Syk in integrin signaling is a recent discovery [40, 42, 53-54]. Inhibition of Syk, or Syk knockdown, suppressed VCAM-1-induced Akt activation and cell adhesion in chronic lymphocytic leukemia cells [40]. Whether integrin-induced Syk activation occurs in MM cells was not known. Here, we show that Syk is activated by reelin-induced β1 engagement in MM cells. Such activation could be reduced or blocked by reelin-specific siRNAs, or by treatment with a β1 inhibitory antibody or Src inhibitor, whereas replenishment of recombinant reelin to siRNA-transfected cells alleviated the inhibition of Syk phosphorylation. This suggests that reelin-induced Syk phosphorylation requires the activation of integrin β1 and Src (Figure 7).

We also showed, for the first time, that reelin-induced integrin β1 activation and subsequent Syk phosphorylation results in STAT3 activation and up-regulation of STAT3 target gene expression. The oncogenic transcription factor STAT3 is often constitutively activated in various types of human cancer, including MM, and is closely associated with cancer cell proliferation and anti-apoptosis [55]. In MM cells specifically, IL-6 is believed to contribute to the activation of Jak2/STAT3 pathway [45]. The phosphorylation of STAT3 by integrin-activated Syk was only recently found in acute myeloid leukemia cells and T-lymphoma cells, but not in activated B lymphoid cells [42-43]. The current study indicates that the plasma cell malignancy is also associated with Syk-activated STAT3 directly downstream of reelin-induced integrin β1 engagement and FAK/Src activation (Figure 7). The activation of STAT3 in MM cells in turn upregulates its target gene expression, including anti-apoptotic members Bcl-2, Mcl-1 and survivin, resulting in improved survival upon Doxorubicin treatment.

It was reported that STAT3 phosphorylation induced by LFA-1 (α_L_β2) cross-linking promoted the migration of T-lymphoma cell line via binding with microtubule-destabilizing protein stathmin [43]. T cell adhesion and the actin cytoskeletal network, however, were not suppressed by STAT3-inhibitory peptide. In our results, reelin-induced MM cell adhesion was also not abolished when STAT3-specific siRNAs were introduced into the cells. Whether integrin β1-induced STAT3 activation facilitates the migration of MM cells awaits further investigation.

Syk is also an upstream regulator of anti-apoptotic PI3K-Akt pathway [56]. Our results revealed that the phosphorylation of Akt at Ser-473 in MM cells upon reelin stimulation could be suppressed by Syk inhibitor (Figure 7). The activation of Akt subsequently upregulated its downstream target, survivin, in reelin-overexpressing MM cells.

Taken together, we demonstrate that reelin promotes MM cell adhesion, survival, and drug resistance via activation of integrin α5β1. The β1 activation subsequently results in phosphorylation of non-receptor tyrosine kinase FAK/Src and Syk, which in turn activates STAT3 and Akt (Figure 7), leading to the promotion of tumor cell survival. In addition, these findings further highlight the therapeutic potential of targeting the reelin/integrin/FAK/STAT3 axis.

## MATERIALS AND METHODS

### Cell lines and primary tumors

The human myeloma cell line (HMCL) RPMI8226 was obtained from the National Platform of Experimental Cell Resources for Sci-Tech (Beijing, China). Other HMCLs including NCI-H929 (shown as H929) and U266 were kindly provided by Prof. Jian Hou from Shanghai Chang Zheng Hospital and Prof. Yu Zhang from Peking University Health Science Center (Beijing, China), respectively. H929, U266, and RPMI8226 cells were cultured in RPMI 1640 (GibCo, Life Technologies, Grand Island, NY, USA) supplemented with 10% or 20% (RPMI8226) fetal bovine serum (Hyclone, ThermoFisher Scientific, Waltham, MA, USA). All the cell cultures were supplemented with 2 mmol/L glutamine and 1% penicillin/streptomycin (GibCo) and were cultured at 37°C in a humidified atmosphere of 5% CO_2_.

Primary MM cells were obtained from the BM aspirates of MM patients at the time of diagnostic procedure. The BM aspirates of 3 healthy donors were also obtained. All the samples were collected after informed consent and in compliance with the Declaration of Helsinki under a protocol approved by the Research Ethics Board in Peking University Health Science Center and People's Hospital. The samples were centrifuged over Ficoll-Paque (Sigma-Aldrich, Oakville, ON, USA) and the isolated mononuclear cells were stained with anti-CD138 antibody. The CD138^+^ cells were sorted using FACS Arial II (BD PharMingen, San Diego, CA, USA). The purity of MM cell populations was >97% when analyzed by flow cytometry.

### Quantitative RT-PCR

Total RNA was isolated from the indicated cells using TRIzol (Invitrogen, Grand Island, NY, USA), according to the manufacturer's instructions. The cDNA was synthesized using the Transcriptor First Strand cDNA Synthesis kit (Tiangen, Beijing, China). Real-time PCR was performed using a PCR Master Mix (Roche, Basel Schweiz, Switzerland) according to the manufacturer's protocol. Quantitative PCR was performed on an iCycler (Bio-Rad Laboratories, Hercules, CA, USA). The primer sequences are shown in Supplemental Table 1 and 2. The PCR conditions were 94°C for 12s, 60/58°C for 12s, and 72°C for 12s. The quantification was based on ΔΔCT calculations and was normalized to GAPDH as loading controls.

### Flow cytometric analysis

Flow cytometric analysis was performed using a FACS Gallios (Beckman Coulter, Indianapolis, IN, USA). Anti-CD138-PE, anti-CD138-APC, and anti-CD29-APC antibodies were purchased from eBioscience (San Diego, CA, USA), PE control mouse IgG antibodies from BioLegend (San Diego, CA, USA), anti-CD29-PE (HUTS-21), anti-AnnexinV-FITC, and anti-AnnexinV-APC antibodies were from BD PharMingen (San Diego, CA, USA).

### Transient transfection

Full-length reelin expression vector, pCrl, was a generous gift from Prof. Tom Curran (The Children's Hospital of Philadelphia, Philadelphia, PA). pcDNA3 was used as a control vector. siRNAs against RELN and APOER2 were purchased from RIBOBIO (Guangzhou, China). MM cells growing at logarithmic phase were transfected with either 10μg pCrl or control vector pcDNA3, or 300pmol reelin-specific siRNA, negative control siRNA (siNC), or a specific control siRNA with nucleic acid mutations around the cleavage site (m-siRNA1) using electroporation (Multiporator, Eppendorf, Hamburg, Germany). The sequences of siRNAs were shown in Supplemental Table 3.

### Cell adhesion assays

A 96-well plate was coated with 40 μg/ml of FN (Sigma), 1 mg/ml of Poly-L-Lysine (Sigma), 1 μg/ml reelin (R&D, Minneapolis, MN, USA), or 5% bovine serum albumin (BSA) in PBS at 37°C for 1 hour. BSA in PBS (1%) was then used to block nonspecific binding sites in the wells at 37°C for 1 h before the experiment. HCMLs or transfected H929 cells were pre-incubated with recombinant reelin protein (1 μg/ml) and/or reelin-specific blocking antibody CR-50 (20 μg/ml, MBL International Corporation, Woburn, MA, USA), and/or the blocking antibody against integrin β1 (P4C10 clone, 20 μg/ml, Merck Millipore, Massachusetts, MA), and/or isotype control (Ctrl) mouse IgG antibodies (20 μg/ml, eBioscience). After 1 hour of pre-incubation at 37°C, HMCLs were washed and resuspended at 5×10^5^/ml in RPMI1640 with 0.1% BSA (adhesion medium). The cells were then added in triplicate to FN-coated 96-well plates at 37°C for 1 hour, and unbound cells were removed by three washes with serum-free RPMI 1640. The attached cell numbers were counted for three microscopic fields (20×) per well and averaged. The attached cells were also stained with crystal violet and measured by colorimetric cell adhesion assay at OD 490 using Multiskan MK3 (ThermoFisher Scientific). For the adhesion assay using Brefeldin A (BFA, BD PharMingen), the transfected H929 cells were treated with BFA for 4 hours before being added to FN-coated wells for the adhesion analysis.

### Integrin activation assay

A 96-well plate was coated with 40 μg/ml of FN at 37°C for 1 hour. After washing, plain HMCLs or HMCLs transfected with pCrl or reelin-specific siRNA were cultured in FN-coated plates containing recombinant reelin (1 μg/ml) and/or CR-50 for 1 hour at 37°C. After one wash with HEPES buffer, anti-CD29-PE (HUTS-21) was applied to the cells for 30–40 min at room temperature. The cells were then analyzed by FACS Arial II. To determine the level of nonspecific binding, the cells were stained in parallel with the PE isotype control mouse IgG antibodies.

### Cell apoptosis assay

To assess the drug-induced apoptosis of HMCLs, H929 cells transfected with pCrl or reelin-specific siRNAs were treated with 2 μmol/L Doxorubicin (Cell Signaling Technology, Danvers, MA, USA) for 24 h in 5% BSA- or 40 μg/ml of FN-coated plate. The recombinant reelin (1 μg/ml) was also added to the culture with siRNA-transfected cells. The cells were then stained with annexin V antibody and the cell viability was then measured by flow cytometry. The cell survival was also assessed by cell counting kit-8 (CCK8) assays (DOJINDO Molecular Technologies, Minato-ku, Tokyo, Japan), according to the manufacturer's instructions. To measure the IC_50_, i.e. the 50% inhibitory concentration, pCrl- or control vector-transfected H929 cells in triplicate were incubated with increasing concentrations of Doxorubicin, cisplatin (Sigma Aldrich, St Louis, MO), and imatinib mesylate (Sigma Aldrich). The cell viability was measured by CCK8 assay. The results were calculated by Probit analysis in SPSS software and the experiments were performed twice.

### Immunoblotting and confocal microscopy

Following incubation under the indicated conditions, including the addition of integrin β1-blocking antibodies (20 μg/ml), control mouse IgG antibodies (20 μg/ml), Src inhibitor PP2 (1 μmol/L, Merck Millipore), Syk inhibitor IV, BAY 61-3606 (1 μmol/L, Merck Millipore), PI3K inhibitor LY 294002 (50 μmol/L, Cell Signaling Technology), MM cells were collected and washed twice with ice-cold PBS, and incubated for 10 minutes at 4°C in Triton X-100 lysis buffer (30 mM Tris-HCl pH7.5, 150 mM NaCl, 25 mM NaF, 1% Triton X-100, 10% glycerol, 2 mM Naorthovanadate). The whole-cell lysates were subjected to 6–8% gradient polyacrylamide gels and transferred to nitrocellulose membrane (Whatman, GE Healthcare Life Sciences, Pittsburgh, PA, USA). The primary antibodies used were anti-Reelin (EPR3330(2)), purchased from Abcam (Cambridge, MA, USA), anti-phospho-FAK (Tyr397), anti-FAK, anti-phospho-JAK2 (Tyr1007/1008), anti-JAK2, anti-phospho-STAT3 (Tyr705), anti-STAT3, anti-phospho-Syk (Tyr525/526), anti-Syk, anti-phospho-Src (Tyr416), anti-Src, anti-phospho-Akt (Ser473), anti-Akt, anti-phospho-ERK1/2 (Thr202/Tyr204), anti-ERK1/2, and anti-GAPDH from Cell Signaling Technology. Goat-anti-mouse IRDye 800CW, Goat-anti-mouse IRDye 800CW (LI-COR Biosciences, Lincoln, NE, USA), anti-mouse IgG HRP conjugate, anti-rabbit IgG HRP conjugate (Promega, Madison, WI, USA) were used as the secondary antibodies. The immunoreactive bands were detected by fluorescence with LiCor Odyssey Gel imaging Scanner or chemiluminescence with ECL detection reagents (ThermoFisher Scientific), and exposed to ImageQuant™ LAS 500 (GE Healthcare Life Sciences).

H929 cells transfected with pCrl or reelin-specific siRNAs were cultured on FN-coated plates overnight and stained with anti-actin (Phalloidin, Fluorescein Isothiocyanate Labeled, Sigma-Aldrich), rabbit anti-phospho-FAK at Tyr861 (Invitrogen), secondary anti–rabbit AlexaFluor 594 (ZSGB-BIO, Beijing, China) and H33324 (DOJINDO Molecular Technologies). A laser-scanning confocal microscope (TCS SP5, Leica, Germany) was used to assess the distribution and intensity of FAK in the cells. The objective lens used is HCX PL APO CS 40×. LAS AF was used to process the images.

### Statistical analysis

Chi-square test, and *t*-test were used to compare the demographic characteristics of patients. The Kaplan-Meier method was used to plot the OS and PFS, which were compared between patients with high and low RELN expression using the log-rank test. A *P*-value of ≤ 0.05 was considered statistically significant. Hazard ratios (HRs) and 95% CIs were generated, with a HR < 1.0 indicating survival benefit (or reduced mortality). The calculations were performed using SPSS 20.0 (SPSS Inc, Chicago, IL).

The data from HMCLs were evaluated by two-tailed Student's *t*-test. All data are presented as mean ± SD. The following terminology is used to denote the statistical significance: *p<0.05, **p<0.01, ***p<0.005, ns, not statistically significant.

## SUPPLEMENTARY FIGURES AND TABLES


