# Supplementary material for: Reelin promotes the adhesion and drug resistance of multiple myeloma cells via integrin β1 signaling and STAT3
Source: Oncotarget. 2016 Feb 3;7(9):9844–58. doi: 10.18632/oncotarget.7151 (PMC4891088; doi:10.18632/oncotarget.7151)
Supplement: Supplementary file 1 [file oncotarget-07-09844-s001.pdf]

SUPPLEMENTARY FIGURES, AND TABLES

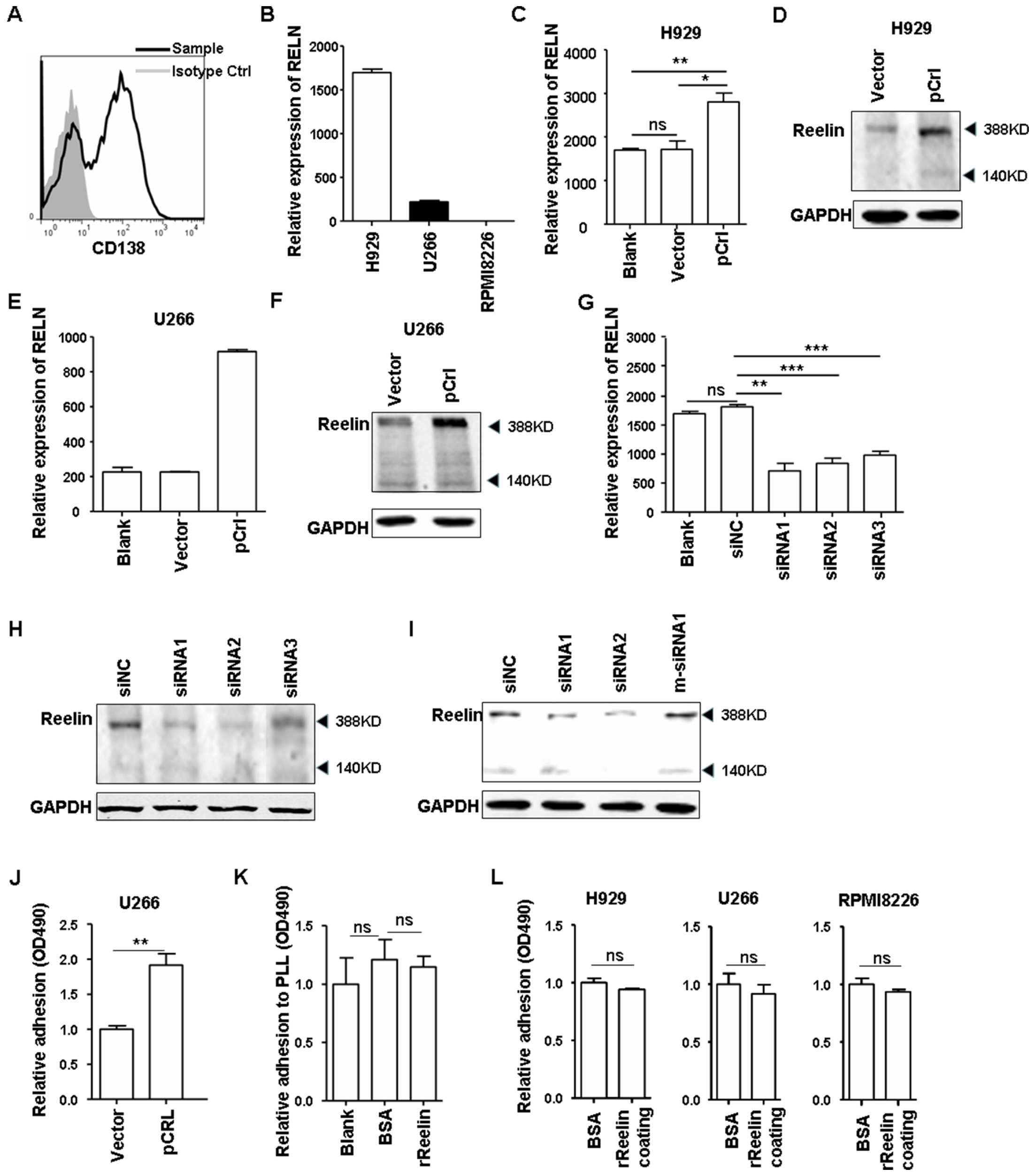

Supplementary Figure S1: Reelin expression in HMCLs. A. CD138 expression in one of the BM samples of MM patients. **B.** The expression of *RELN* mRNA in H929, U266, and RPMI8226 cells, as assessed by quantitative RT-PCR. The quantification was based on ΔΔCT calculations and was normalized to GAPDH. **C-F.** Overexpression of reelin in H929 and U266 cells. H929 and U266 cells were transfected with 10 µg reelin expressing plasmid pCrl or control plasmid pcDNA3 (vector). The cells were harvested 24 or 40 hours later, and the mRNA expression of reelin was analyzed by quantitative RT-PCR (C and E); protein expression was analyzed by western blotting (D and F). **G-I.** Knockdown of reelin in H929 cells. H929 cells were transfected with 300 pmol reelin-specific or control siRNA (siNC and m-siRNA1) for 24 or 40 hours. The cells were harvested and the mRNA (G) and protein expressions (H-I) of reelin were determined. **J.** Overexpression of reelin promotes U266 cell adhesion to FN. **K.** Reelin does not promote cell adhesion to poly-L-Lysine. H929 cells pre-treated with rReelin (1 µg/ml) were seeded onto Poly-L-Lysine (1 mg/ml)-coated plates. Cell adhesion was analyzed colorimetrically 1 hour later. **L.** HMCLs do not show adhesion to reelin-coated plates. H929, U266, and RPMI8226 cells were cultured in rReelin (1 µg/ml)-coated plates for an hour and cell adhesion was analyzed colorimetrically.

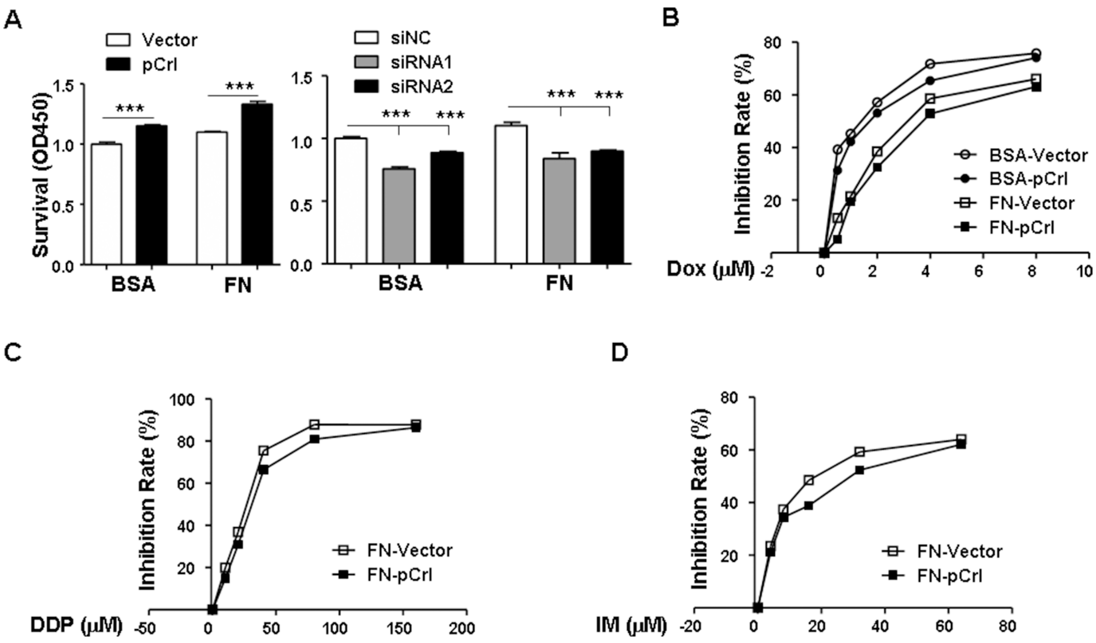

Supplementary Figure S2: Reelin promotes the survival of HMCLs. A. Reelin protects H929 cells from Doxorubicin induced apoptosis. H929 cells were transfected with pCrl or reelin-specific siRNAs for 40 hours. The cells were then treated with Dox (2μM) in the presence of 5% BSA or 40 μg/ml of FN. Cell apoptosis was analyzed 24 hours later by CCK8 method. **B.** IC_{50} of Dox for pCrl- or control vector-transfected H929 cells in the presence or absence of FN. CCK8 method was used to analyze cell viability 24 hours after incubation. **C.** IC_{50} of cisplatin (DDP) for H929 cells in the presence of FN. **D.** IC_{50} of imatinib mesylate (IM) for H929 cells in the presence of FN.

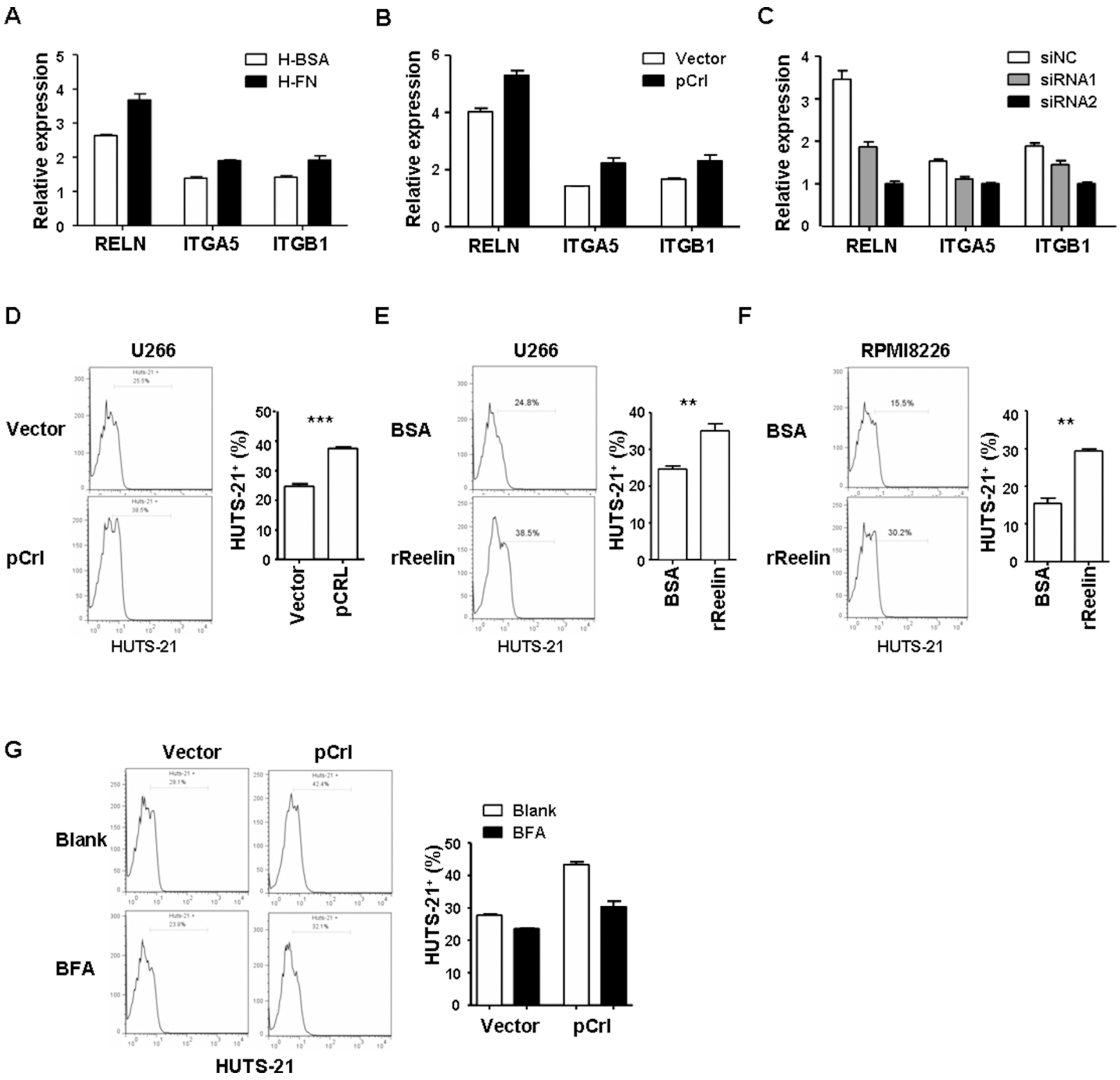

Supplementary Figure S3: Changes in reelin levels alter integrin expressions and activation in H929 cells. A. The adhesion of H929 cells to FN increases the mRNA levels of reelin, integrin α5 and β1. **B-C.** Overexpression (B) or knockdown (C) of reelin alters the transcription level of integrin α5 and β1. H929 cells were transfected with 10 µg reelin expressing plasmid pCrl or control plasmid pcDNA3 (vector), 300 pmol reelin-specific or control siRNA (siNC) for 40 hours. All mRNA expressions were analyzed by quantitative RT-PCR. **D.** Overexpression of reelin promotes integrin β1 activation in U266 cells in the presence of FN. **E-F.** Pretreatment of rReelin for 1 hour promotes integrin β1 activation in U266 cells (E) and RPMI8226 cells (F) in the presence of FN. **G.** Reelin secretion is required to promote the activation of integrin β1. The pCrl-transfected cells were treated with Brefeldin A (BFA) for 4 hours before flow cytometry analysis. The BFA-treated and non-treated (blank) HUTS-21 expression in the vector or pCrl-transfected H929 cells is shown on the left and the mean percentages of HUTS-21^{+} cells in various cell groups from two independent experiments are shown on the right.

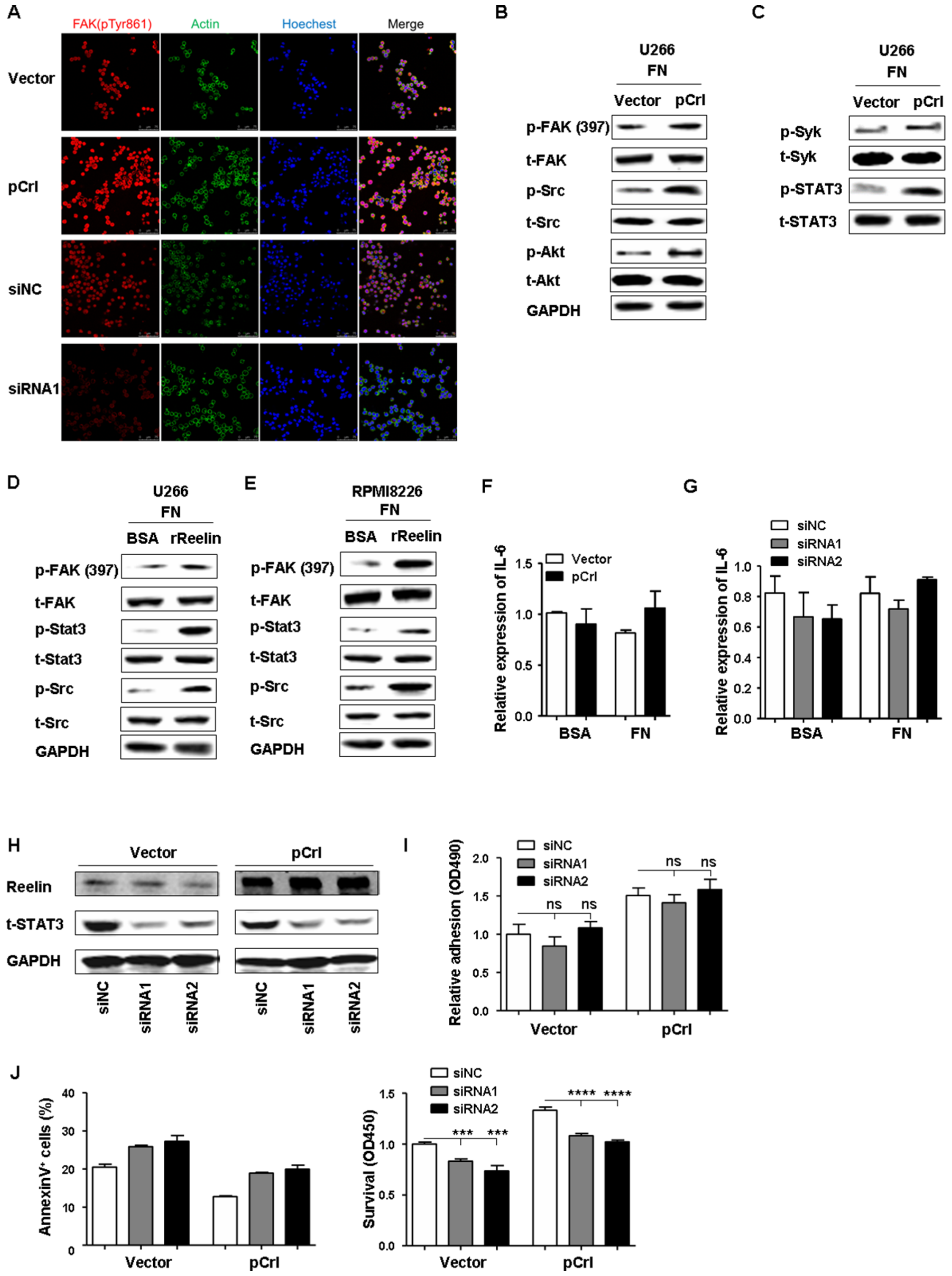

Supplementary Figure S4: Reelin expression in MM cells activates FAK/Src/Syk/STAT3 signaling pathway and cell survival but does not affect IL-6 production. A. The phosphorylation of FAK at Tyr 861 is altered by reelin overexpression and knockdown. H929 cells were transfected with pCrl or control plasmid, or reelin-specific or control siRNA for 40 hours before being cultured in FN-coated plates overnight. The cell lysates were then subjected to antibodies against phospho FAK Tyr 861 (red), actin (green). Nuclei were stained with Hoechest 33342. The cells were analyzed by laser-scanning confocal microscopy (40x). **B.** Reelin overexpression promotes the activation of FAK, Src, and Akt in U266 cells in the presence of FN. **C.** Overexpression of reelin promotes the activation of Syk and STAT3 in U266 cells. **D-E.** The addition of recombinant reelin protein promotes the activation of FAK, STAT3, and Src in U266 (D) and RPMI8226 (E) cells in the presence of FN. **F-G.** Altered reelin expression does not affect IL-6 production by H929 cells. H929 cells were transfected with pCrl and the vector control (F) or reelin-specific siRNA and siRNA control (G) for 40 hours. Total RNAs were extracted and the expression of IL-6 was analyzed by quantitative RT-PCR. **H.** Knockdown of STAT3 in H929 cells. H929 cells were transfected with pCrl/vector control and STAT3 siRNAs for 40 hours. The level of total STAT3 protein 24 hours after siRNA transfection is shown. **I.** Reelin's promotion of H929 cell adhesion to FN is not affected by STAT3 knockdown. H929 cells were co-transfected with pCrl and STAT3-specific siRNAs (or control siRNA, siNC) or pcDNA3 with STAT3-specific siRNAs (or siNC). Forty hours later, the cells were cultured in FN-coated plates for one hour. Cell adhesion was analyzed by colorimetric cell adhesion assay. **J.** Reelin-mediated promotion of cell survival is abolished when knocking down STAT3 expression. The STAT3 siRNA and pCrl/vector co-transfected cells were treated with Dox (2 µM) in FN-coated plates. The cell apoptosis was analyzed 24 hours later by Annexin V staining (left) and CCK8 (right) method. The experiments were repeated for two or three times and similar results were seen.

Supplementary Table S1: Primers used in qRT-PCR analyses (5'-3')

RELN	forward	GATGGGCGGCGTCAGCTAAT
	reverse	GGCTCTGCACGTGCTCAGAA
GAPDH	forward	ACCCACTCCTCCACCTTTGA
	reverse	CTGTTGCTGTAGCCAAATTCGT
ITGB1	forward	CCGGTCCAACCTGATCCTGTG
	reverse	ATTCCAGCAACCACACCAGCT
ITGA3	forward	ACTCGGTCGCCCTCCATCGG
	reverse	CAGTCATCCTTGTGGGCAGT
ITGA5	forward	GGCAGCTATGGCGTCCCACTGTGG
	reverse	GGCATCAGAGGTGGCTGGAGGCTT
BAD	forward	TGAGTGACGAGTTTGTGGACTCCT
	reverse	CTGGAGCTTTGCCGCATCT
BAX	forward	GCGAGTGTCTCAAGCGCATC
	reverse	CCAGTTGAAGTTGCCGTCAGAA
BCL2	forward	GATTGATGGGATCGTTGCCTTA
	reverse	CCTTGGCATGAGATGCAGGA
BCL2L1	forward	CTTGCAGTTCAGCACCACCCTA
	reverse	GTGAGGCAGCTGAGGCCATAA
PIM1	forward	ACGCTTGCTCTGTTTGTGG
	reverse	CTGGAAGGCACACCATCC
MCL1	forward	TAAGGACAAAACGGGACTGG
	reverse	GCTCCTACTCCAGCAACACC
BIRC5	forward	CCACCGCATCTCTACATTCAA
	reverse	CTCTATGGGGTCGTCATCTGG
IL6	forward	AAATCATCACTGGTCTTTTGGAG
	reverse	GGTCAGGGGTGGTTATTGC

Supplementary Table S2: Primers used in RT-PCR analyses (5'-3')

DAB1(1)	forward	AAGTAGCTGTGAAAACCAGCG
	reverse	CCCGGTGATCTGTAATGTCCT
DAB1(2)	forward	AGGTCAGGATCGCAGTGAAGC
	reverse	GGAACGAGCGCCAGCAACAAC
VLDLR	forward	CCTAGCTCATCCTCTTGCACTAAC
	reverse	TGGCACCATAGACTGCTTCATT
ApoER2	forward	ACAATATTGAATGGCCCAACG
	reverse	TCAATGCTGGACAGTTGGTGTA
EPHB2	forward	GAAGGAGCTCAGTGAGTACAACG
	reverse	GCACCTGGAAGACATAGATGG
EFNB2	forward	GCAAGTTCTGCTGGATCAAC
	reverse	AGGATGTTGTTCCCCGAATG

Supplementary Table S3: Sequences of siRNAs

siRNAs	Sense strand (5'-3')
Reelin siRNA1	CCAGCAUCAUCGUGUUAUAdTdT
Reelin siRNA2	GGCGAUUGAUAAUGUUGUAdTdT
Reelin siRNA3	CGAGGACACUGCACUCUAUdTdT
Reelin m-siRNA1	CCAGCAGACGAUUGUUAUAdTdT
STAT3 siRNA1	GAGAUUGACCAGCAGUAUAdTdT
STAT3 siRNA2	CCAACAAUCCCAAGAAUGUdTdT

The difference between Reelin siRNA1 and m-siRNA1 is underlined.
